# Surgical Reconstruction of the Lateral Patellofemoral Restraint Complex for Medial Patellar Instability: A Systematic Review of Techniques and Clinical Outcomes

**DOI:** 10.1177/23259671261486592

**Published:** 2026-10-01

**Authors:** Krishnateja Narayana, Helena Son, Marc Daniel Bouchard, Prushoth Vivekanantha, Janan Ashique, Ashraf Hantouly, Darren de SA, Jeffrey Kay, Olufemi R. Ayeni

**Affiliations:** * Michael G. DeGroote School of Medicine, McMaster University, Hamilton, Ontario, Canada; † McMaster University, Division of Orthopaedic Surgery, Hamilton, Ontario, Canada; Investigation performed at McMaster University, Hamilton, Ontario, Canada

**Keywords:** lateral patellofemoral ligament, lateral patellofemoral restraint complex, lateral patellotibial ligament, medial patellar instability, reconstruction, systematic review

## Abstract

**Background:**

Medial patellar instability is an uncommon, frequently iatrogenic complication after lateral retinacular release or other lateral-sided procedures. Multiple reconstructive techniques for the lateral patellofemoral restraint complex (LPFRC) have been described, but evidence remains heterogeneous.

**Purpose:**

To characterize surgical techniques for LPFRC reconstruction and summarize associated clinical outcomes.

**Study Design:**

Systematic review; Level of evidence, 4.

**Methods:**

A PRISMA (Preferred Reporting Items for Systematic Reviews and Meta-Analyses)-compliant systematic review of MEDLINE, EMBASE, Scopus, and Web of Science was conducted from database inception through January 11, 2026, for studies describing surgical reconstruction of the LPFRC for medial patellar instability. Two reviewers independently extracted data on operative characteristics, rehabilitation, and clinical outcomes; the methodological quality was assessed using the MINORS (Methodological Index for Non-Randomized Studies) checklist. Findings were synthesized descriptively without meta-analysis due to heterogeneity in techniques and reporting.

**Results:**

Twenty studies were included: 6 case series, 5 case reports, and 9 technical notes. Iatrogenic instability after prior lateral-sided surgery occurred in 15 studies (75%). Fourteen studies described anatomic ligament reconstruction, 5 pedicled soft-tissue reconstruction, and 3 retinacular imbrication or sling procedures (not mutually exclusive). MINORS scores among 6 eligible noncomparative studies ranged from 6 to 11 of 16 (mean, 8.8). Clinical outcomes were reported in 10 studies, with follow-up ranging from 12 to 99 months. Six studies reported numeric pre- and postoperative scores: the Knee injury and Osteoarthritis Outcome Score improved from 34.4 to 69.5 points (*P* < .0001), the Lysholm score from 45.6 to 71.9 points (*P* < .001), and, in another cohort, the Lysholm score improved from 36.4 to 86.1 while the visual analog scale-pain score decreased from 7.6 to 1.9 (*P* < .001). Across the outcome-bearing studies, 7 recurrent instability events were reported, concentrated in 2 studies. Complication reporting was sparse and inconsistent.

**Conclusion:**

LPFRC reconstruction is described using technically diverse, predominantly low-level, noncomparative evidence. Although individual studies reported postoperative improvements, small cohort sizes, inconsistent reporting of outcomes, and methodological limitations preclude comparisons between techniques or definitive conclusions about efficacy. Prospective comparative studies with standardized outcome reporting are needed.

Patellar instability is a multifactorial condition traditionally thought of as a laterally based pathology, most commonly presenting as recurrent lateral patellar subluxation or dislocation.^37^ In contrast, medial patellar instability is a rare but clinically significant entity, as the vast majority of patellar instability events occur in the lateral direction.^15,18,33^ Medial patellar instability ranges from subluxation to dislocation. In the literature these terms are often used inconsistently, with most studies relying on clinical assessment rather than standardized quantitative thresholds. Although some degree of medial patellar translation may be observed in physiologically lax individuals, pathologic instability is typically characterized by symptomatic apprehension, abnormal patellar tracking, and a history of lateral-sided disruption. As such, the diagnosis of medial patellar instability remains primarily clinical and context-dependent. If left unaddressed, medial instability can lead to pain and functional limitation.^15,18^ The lateral patellofemoral restraint complex (LPFRC) comprises the lateral patellofemoral ligament (LPFL), lateral patellotibial ligament (LPTL), lateral retinaculum, and associated capsuloligamentous structures.^10^ These structures resist excessive medial translation of the patella and contribute to dynamic patellar stability throughout knee flexion.^11^ Disruption or insufficiency of the LPFRC, whether iatrogenic or traumatic, can result in symptomatic medial instability that is often refractory to nonoperative treatment.

A variety of surgical strategies have been described to restore lateral restraint, including anatomic LPFL or LPTL reconstruction, lateral retinacular reconstruction, pedicled soft-tissue augmentation, and sling-based procedures.^6,18,21,26,27,33^ However, these techniques vary substantially with respect to graft selection, fixation method, anatomic fixation site, tensioning protocol, and knee flexion angle at fixation. Given the rarity of medial patellar instability requiring surgical restoration of the lateral restraints, the available literature largely comprises small case series, case reports, and technical notes, resulting in considerable heterogeneity in both surgical approaches and outcome reporting. Previous systematic reviews and studies have primarily focused on clinical outcomes after surgical management of medial patellar instability.^20,22,25,33,35^ While these reviews demonstrate generally favorable short- to mid-term results, they do not systematically classify reconstructive constructs or synthesize variability in operative technique. Consequently, there remains no structured framework for understanding how LPFRC reconstructions are performed or how technical variability may influence reported outcomes. Accordingly, this systematic review sought to answer 2 questions: (1) What surgical techniques have been described to restore the LPFRC in patients with medial patellar instability, and how can these techniques be organized according to their anatomic target and reconstructive strategy? and (2) What clinical outcomes and complications have been reported after these procedures?

## Methods

This systematic review was conducted in accordance with the PRISMA (Preferred Reporting Items for Systematic Reviews and Meta-Analyses) guidelines.^16^ As this study synthesized previously published data, institutional review board approval and patient consent were not required. This study was not registered with the International Prospective Register of Systematic Reviews (PROSPERO).

### Search Criteria

Studies were eligible for inclusion if they (1) involved human patients with medial patellar instability (medial subluxation or dislocation), including iatrogenic cases after prior patellofemoral procedures; (2) described surgical repair, reconstruction, or restoration of the LPFRC to re-establish lateral patellar restraint; (3) reported operative technique details (eg, graft type, fixation method or location, tensioning parameters, or key technical steps); and (4) were clinical studies such as case reports, case series, or retrospective or prospective cohort studies. No restrictions were placed on the year of publication.

Studies were excluded if they focused on lateral patellar instability without a medial instability cohort, evaluated medial-sided reconstruction alone, or investigated nonreconstructive procedures (eg, isolated retinacular release, decompression, or denervation) that did not involve repair, reconstruction, or restoration of the LPFRC. Narrative reviews, systematic reviews, editorials, letters, expert opinion articles, conference abstracts without full text, non-English publications, and inaccessible full-text articles were also excluded.

### Search Strategy and Study Screening

Electronic database searches were performed from database inception through January 11, 2026, across MEDLINE, EMBASE, Scopus, and Web of Science. The search strategy combined controlled vocabulary (eg, MeSH and Emtree terms) and free-text keywords related to “*lateral patellofemoral restraint structures,” “LPFL,” “LPTL,” “lateral retinacular reconstruction,”* and “*medial patellar instability.”* The complete line-by-line search strategy for each database is provided in Supplementary Material 1. Reference lists of included abstracts and relevant reviews were manually screened to identify additional eligible studies. All results were imported into Covidence (Veritas Health Innovation) where duplicates were automatically removed.

Two authors (K.N. and H.S.) independently screened title/abstracts and full-text articles using eligibility criteria. Inter-reviewer agreement was calculated using Cohen’s kappa. Agreement levels were predefined as follows: κ = 0.91-0.99, almost perfect; κ = 0.71-0.90, substantial; κ = 0.61-0.70, high; κ = 0.41-0.60, moderate; κ = 0.21-0.40, fair; and κ ≤ 0.20, no agreement.^14^ Disagreements in the title/abstract screening stage were advanced to full-text review to avoid any premature exclusions. Any full-text disagreements were resolved through direct discussion between a third senior author (M.D.B.).

### Quality Assessment

Methodological quality of included clinical studies was independently assessed by 2 reviewers (K.N. and H.S.) using the Methodological Index for Non-Randomized Studies (MINORS) criteria.^34^ The MINORS instrument can be applied to both comparative studies (12 items; maximum score 24) and noncomparative studies (8 items; maximum score 16). Because all outcome-bearing studies in the present review were noncomparative in design, the noncomparative version of the MINORS tool was applied. Each item was scored as 0 (not reported), 1 (reported but inadequate), or 2 (reported and adequate) according to established guidelines. Any discrepancies in scoring were resolved through discussion until consensus was achieved. Case reports were not formally scored using MINORS because they represent individual patient observations rather than cohort-level studies. Instead, these studies underwent a structured descriptive appraisal focused on the clarity of patient selection, surgical indication, operative technique, outcome definition, follow-up duration, and reporting of complications. Technical notes were not formally assessed using MINORS because their primary purpose was procedural description and they did not represent formal cohort studies. Any limited clinical observations reported within technical notes were appraised descriptively.

### Data Extraction

Two authors (K.N. and H.S.) performed data extraction, with the included studies divided between reviewers using a standardized, pilot-tested spreadsheet (Google Sheets; Google LLC). Extracted entries were cross-checked for accuracy and completeness, and any uncertainties or discrepancies were discussed and resolved through consensus.

When a technical note and a separate clinical study (eg, a case report or case series) described the same surgical technique by the same author group, both publications were included for completeness. However, technical notes were used exclusively for procedural characterization, while clinical outcome data were extracted only from the corresponding outcome-bearing study to avoid duplication of patient data. Technical notes primarily informed the procedural synthesis and classification. When limited clinical outcome data were reported, these findings were extracted and summarized descriptively but were not treated as equivalent to outcomes from formal clinical cohort studies.

The following variables were extracted from each study: author and year of publication, country of study, study design and level of evidence, sample size and patient characteristics, surgical indication, LPFRC structure addressed, reconstruction type and whether the technique represented a primary description or application of a previously described method, graft type, fixation method and anatomic fixation site, postoperative rehabilitation protocols, follow-up duration, and postoperative outcomes, including recurrent medial instability and patient-reported outcome measures (PROMs).

### Data Synthesis

Outcome-bearing studies were categorized according to the nature of reported outcome data. Studies reporting extractable numeric PROM or pain scores were summarized separately from those reporting categorical or narrative outcomes to preserve methodological consistency and avoid inappropriate comparisons across heterogeneous reporting formats. Categorical variables were summarized using counts and percentages, whereas continuous variables were summarized using means, medians, and ranges, as appropriate.

Given the heterogeneity in surgical techniques, outcome reporting formats, and PROM instruments across studies, quantitative meta-analysis was not performed. No inferential statistics were conducted. Instead, findings were synthesized descriptively, grouped according to reconstruction technique, and stratified by quantitative versus qualitative outcome reporting. Any *P* values presented were extracted as reported by the individual included studies and were not calculated as part of the present review.

## Results

### Search Results and Screening Process

A total of 2365 studies were identified through database searching, including Web of Science (n = 720), Scopus (n = 613), Embase (n = 574), and MEDLINE (n = 457), with one additional record identified through reference list screening. After removal of 1294 duplicate records using Covidence, 1071 unique studies underwent title and abstract screening. Inter-reviewer agreement at this stage was fair (κ = 0.30). Of these, 1019 studies were excluded based on irrelevance to the review topic. A total of 52 full-text articles were assessed for eligibility, with substantial agreement between reviewers at this stage (κ = 0.72). Ultimately, 20 studies met the inclusion criteria and were included in the final analysis (Figure 1).

**Figure 1.**
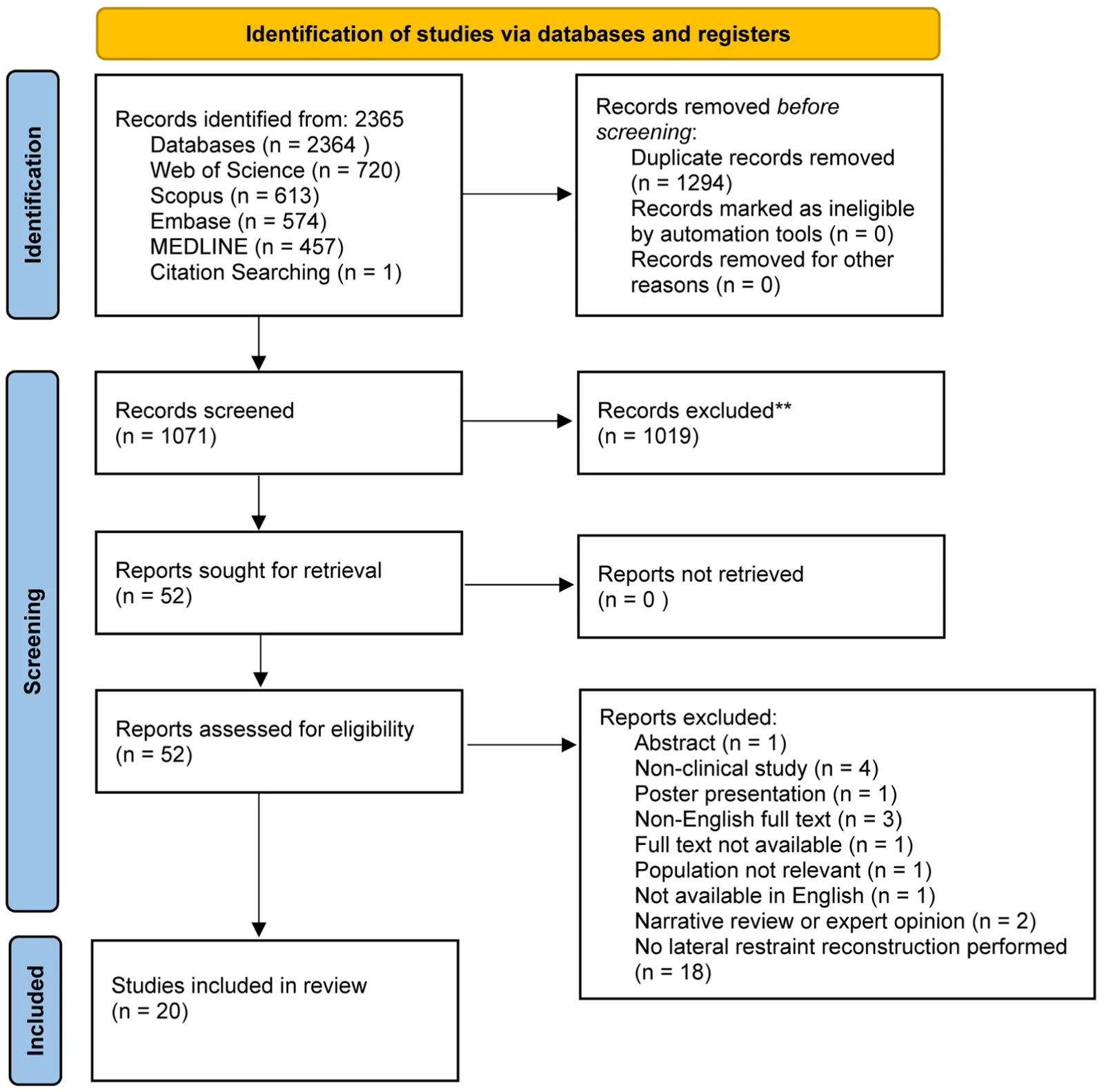
PRISMA flow diagram of study selection. PRISMA, Preferred Reporting Items for Systematic Reviews and Meta-Analyses.

### Study and Patient Characteristics

A total of 20 studies (1994-2025) met the inclusion criteria, most originating from the United States (n = 13), with the remainder from Spain, Japan, the United Kingdom, Turkey, Switzerland, and India (Table 1). The evidence base consisted entirely of lower-level, noncomparative literature: 6 (Level 4) case series, 5 case reports (Level 4 and 5), and 9 (Level 5) technical notes; no randomized or comparative cohort studies were identified.

**Table 1:** Study Characteristics of Included Articles on LPFRC Reconstruction for Medial Patellar Instability^*a*^

Author	Year	Country	Study Design	LOE	Sample Size (Patients/Knees)	Age, Years (Range)	% Female (n/N)	Follow-up (Months)
Abhaykumar et al^1^	1999	United Kingdom	Case series	4	3/4	NR (27-39)	100 (3/3)	(60-84)
Aksahin et al^2^	2010	Turkey	Case report	5	1/1	21	0 (0/1)	12 (NR)
Arora et al^3^	2024	India	Technical note	5	N/A	N/A	N/A	12 (NR)
Beckert et al^5^	2016	United States	Retrospective case series	4	17/19	29.5 (15.4-54.4)	94.7 (18/19 knees)^*b*^	24.6 (NR)
Borbas et al^6^	2014	Switzerland	Case report	5	1/1	62 (NR)	100 (1/1)	12 (NR)
Decilveo et al^9^ (2025a)	2025	United States	Case report	5	1/1	35	100 (1/1)	24 (NR)
Decilveo et al^8^ (2025b)	2025	United States	Technical note	5	N/A	N/A	N/A	N/A
Hughston et al^12^	1996	United States	Retrospective case series	4	63/65	29.2 (14-49)	79 (50/63)	53.7 (24-99)
Kerzner et al^13^	2022	United States	Technical note	5	N/A	N/A	N/A	N/A
Moatshe et al^21^	2017	United States	Case series	4	13/13	31.3 (15-46)	84.6 (11/13)	46.5 (24-60)
Nonweiler et al^22^	1994	United States	Case series	4	5/5	NR (19-28)	80 (4/5)	39.6 (12-84)
Parvaresh et al^23^	2021	United States	Technical note	5	N/A	N/A	N/A	N/A
Sanchis-Alfonso et al^26^ (2015a)	2015	Spain	Technical note	5	N/A	N/A	N/A	N/A
Sanchis-Alfonso et al^27^ (2015b)	2015	Spain	Retrospective case series	4	17/17	28 (16-43)	76.5 (13/17)	52.8 ± 23.6 (24-96)
Saper et al^28^	2018	United States	Technical note	5	N/A	N/A	N/A	N/A
Saper et al^30^ (2014a)	2014	United States	Case report	4	1/1	14	100 (1/1)	12 (NR)
Saper et al^29^ (2014b)	2014	United States	Technical note	5	3/3	N/A	NR	12 (NR)
Sawyer et al^31^	2014	United States	Technical note	5	N/A	N/A	N/A	N/A
Teitge et al^35^	2004	United States	Technical note	5	60/60	N/A	N/A	N/A
Udagawa et al^36^	2014	Japan	Case report	4	1/1	20	100 (1/1)	24 (NR)
**Total**	-	United States (n = 13), Spain (n = 2), United Kingdom (n = 1), Turkey (n = 1), Switzerland (n = 1), India (n = 1), Japan (n = 1)	Technical notes (n = 9), case reports (n = 5), case series (n = 3), retrospective case series (n = 3)	4 (n = 7), 5 (n = 13)	-	-	-	-

a Follow-up values were standardized to months for consistency across studies. “Not applicable” refers to technical notes that do not include patient cohorts. LOE, level of evidence; LPFRC, lateral patellofemoral restraint complex; n/N, number of patients or knees over total sample size; NR, not reported.

b Beckert et al^5^ reported sex distribution by knees rather than by patients.

Among the 11 studies reporting clinical cohorts, sample sizes ranged from 1 to 63 patients (1-65 knees), with a median cohort size of 3 patients (range, 1-63). The mean age, when reported, ranged from 14 to 62 years, and most cohorts were predominantly female, although sex reporting was inconsistent. The median follow-up across outcome-bearing studies was 24 months (range, 12-84 months). A full breakdown of study characteristics is provided in Table 1.

#### Methodological Quality and Risk of Bias

Methodological quality was formally assessed with the MINORS score in the 6 noncomparative clinical studies reporting patient-level outcomes; scores ranged from 6 to 11 of a possible 16 (mean, 8.8), reflecting substantial methodological limitations and at least moderate concern for bias. All outcome-bearing studies were uncontrolled, and most cohorts were small, increasing susceptibility to selection bias and limiting the precision and generalizability of the reported findings. No randomized or comparative cohort studies were identified. (Supplementary Material 2). Technical notes were excluded from demographic summaries because they did not provide sufficiently complete cohort-level demographic data. Limited descriptive clinical observations reported within technical notes were summarized separately in the qualitative outcome synthesis. The 5 case reports were not amenable to MINORS scoring and instead underwent a structured descriptive appraisal (Supplementary Material 3).

### Surgical Indications and Targeted Anatomy

Iatrogenic instability after prior lateral-sided surgery, most commonly lateral retinacular release, was the most predominant indication, reported in 15 of 20 studies, and was diagnosed clinically based on medial translation and/or apprehension; imaging criteria were inconsistently reported (Table 2). Two studies described mixed iatrogenic and traumatic/spontaneous etiologies,^12,28^ and 4 described primary, noniatrogenic instability without prior lateral release.^2,23,28,29^ Based on these findings, indications across the literature can be broadly categorized into (1) isolated iatrogenic medial instability after prior lateral-sided procedures; (2) mixed etiologies involving both prior surgery and traumatic or spontaneous instability; and (3) primary, noniatrogenic medial instability without prior lateral release.

**Table 2:** Indications for LPFRC Reconstruction and Targeted Lateral Restraint Structures^*a*^

Study	Surgical Indication	Reported Cause of Instability	Cause	Reported Diagnostic Criteria	Target Structure
Abhaykumar et al^1^	Recurrent medial patellar dislocation	Tibial tubercle transfer with previous lateral retinacular release	Iatrogenic	Clinical examination	Lateral retinaculum
Aksahin et al^2^	Spontaneous medial patellar subluxation refractory to physical therapy	None	Noniatrogenic	Clinical examination and conventional radiographs	LPTL
Arora et al^4^	Medial patellar instability (often postlateral release)	Prior lateral retinacular release (commonly reported)	Iatrogenic	NR	LPFL
Beckert et al^5^	Medial patellar instability after lateral release	Lateral retinacular release	Iatrogenic	Physical examination with medial instability/apprehension testing	LPFL
Borbas et al^6^	Medial instability after arthroplasty and realignment	TKA + lateral retinacular release + TTO	Iatrogenic	Clinical examination with stress radiography/CT	LPFL
Decilveo et al^9^ (2025a)	Revision medial instability	Capsular release + failed LPFL reconstruction	Iatrogenic	Clinical examination with CT/MRI/radiographic evaluation	LPFL + LPTL
Decilveo et al^8^ (2025b)	Revision medial instability	Failed LPFL reconstruction (prior ACLR + TTO history noted)	Iatrogenic	NR	LPFL + LPTL
Hughston et al^12^	Symptomatic medial patellar subluxation	Predominantly lateral retinacular release; some traumatic/surgical causes	Mixed	Passive medial patellar subluxation/dislocation reproducing symptoms	LPTL ± lateral retinaculum
Kerzner et al^13^	Chronic medial instability	Lateral retinacular release	Iatrogenic	Clinical history and physical examination	LPFL
Moatshe et al^21^	Symptomatic medial patellar instability after prior lateral release	Lateral retinacular release	Iatrogenic	Clinical examination with dynamic medial patellar subluxation; reverse McConnell taping	LPTL
Nonweiler et al^22^	Medial subluxation	Isolated lateral retinacular release	Iatrogenic	Clinical examination with medial apprehension and gravity subluxation tests	Lateral retinaculum
Parvaresh et al^23^	Bidirectional patellar instability refractory to nonoperative care	None specified (congenital/bidirectional instability cohort)	Noniatrogenic	NR	LPFL + lateral retinaculum
Sanchis-Alfonso et al^26^ (2015a)	Iatrogenic medial instability	Lateral retinacular release	Iatrogenic	Reverse McConnell taping; stress radiography/CT	Lateral retinaculum (deep transverse layer)
Sanchis-Alfonso et al^27^ (2015b)	Iatrogenic medial instability	Lateral retinacular release	Iatrogenic	Clinical examination with Fulkerson relocation test; stress radiography/CT	Lateral retinaculum (deep transverse layer)
Saper et al^28^	Isolated medial patellar subluxation	None or prior lateral release (mixed cohort)	Mixed	Clinical examination	LPFL
Saper et al^30^ (2014a)	Medial patellar subluxation without prior release	None	Noniatrogenic	NR	LPFL
Saper et al^29^ (2014b)	Isolated medial patellar subluxation	None specified	Noniatrogenic	NR	LPFL
Sawyer et al^31^	Medial patellar instability	Lateral retinacular release	Iatrogenic	Clinical examination with reverse McConnell taping	LPTL
Teitge et al^35^	Medial dislocation/subluxation	Lateral retinacular release	Iatrogenic	Stress radiography	LPFL
Udagawa et al^36^	Medial instability after prior reconstruction	Lateral retinacular release + malpositioned/overtensioned MPFL reconstruction	Iatrogenic	Clinical examination with stress radiography	Lateral retinaculum
Total	-	-	Iatrogenic (n = 14), noniatrogenic (n = 4), mixed (n = 2)		Isolated LPFL (n = 8), isolated LPTL (n = 3), LPFL + LPTL (n = 2), lateral retinaculum (n = 7)^*b*^

a ACLR, anterior cruciate ligament reconstruction; CT, computed tomography; LPFL, lateral patellofemoral ligament; LPTL, lateral patellotibial ligament; MPFL, medial patellofemoral ligament; MRI, magnetic resonance imaging; NR, not reported; TTO, tibial tubercle osteotomy.

b Includes isolated lateral retinacular reconstruction and hybrid constructs incorporating lateral retinacular augmentation.

LFPL-based reconstruction was the most frequently targeted structure, described in 11 studies,^§^ followed by lateral retinacular reconstruction (7 studies),^1,12,22,23,26,27,36^ isolated LPTL reconstruction (3 studies),^2,21,31^ and combined LPFL/LPTL reconstruction (2 studies, generally in revision settings).^8,9^ Notably, despite variability in reported causes, these surgical strategies were consistently directed toward restoration of lateral restraint, with relatively limited reporting of isolated revision of medial-sided procedures. Overall, substantial heterogeneity was observed in both the underlying cause and the specific anatomic targets addressed across the included literature.

### Surgical Technique Characteristics

Three broad reconstructive approaches were described across studies: (1) anatomic ligament reconstruction using graft-based constructs to restore native lateral restraint anatomy; (2) pedicled soft-tissue–based reconstructions preserving native distal iliotibial band (ITB) attachment; and (3) retinacular repair, with or without imbrication, or sling-type procedures (Figure 2). These categories were not mutually exclusive, as several studies incorporated hybrid constructs or augmentation techniques.

**Figure 2.**
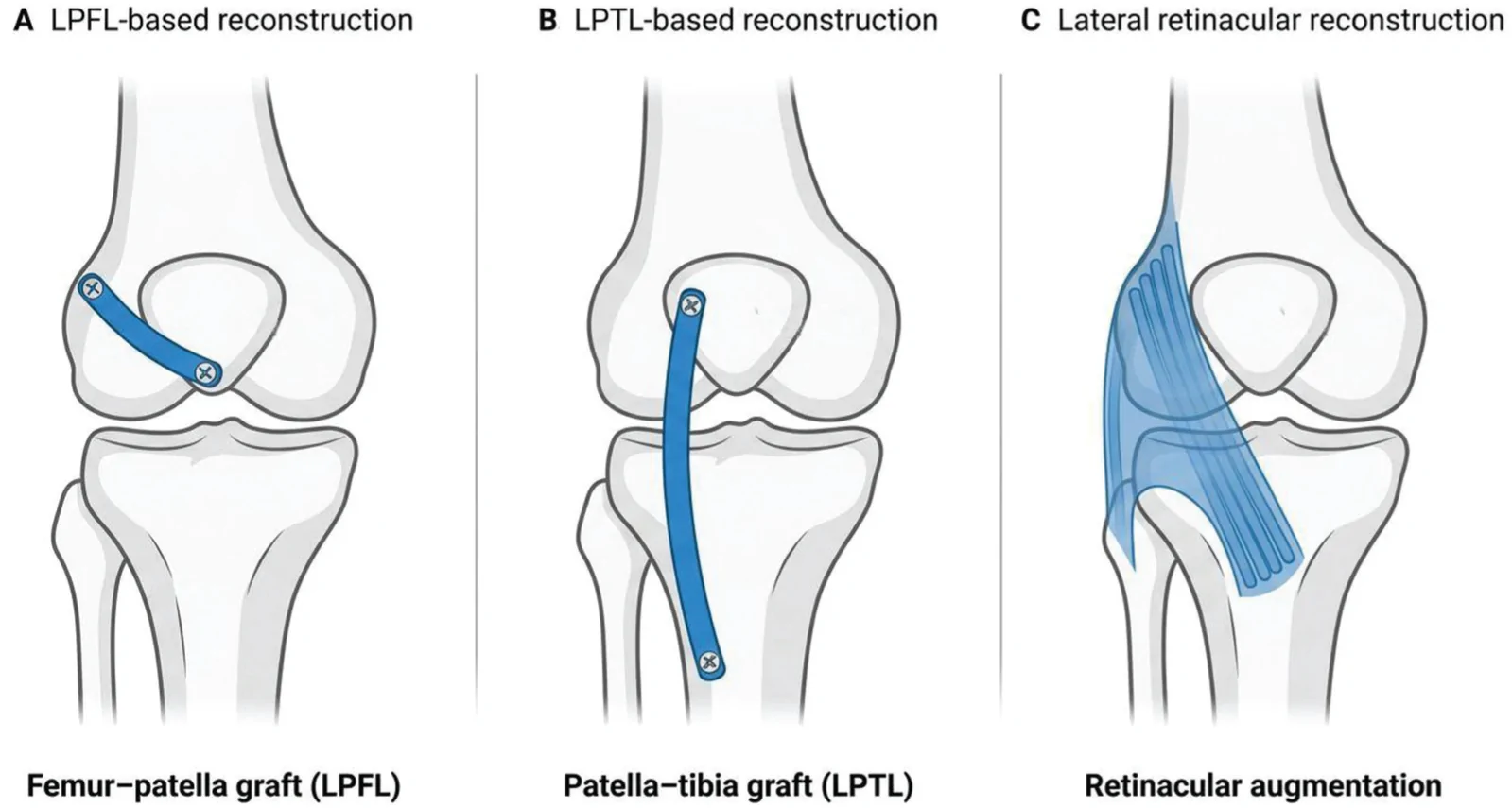
Schematic illustration of surgical techniques for restoration of lateral patellar restraint. (A) LPFL reconstruction demonstrating a femur-to-patella graft restoring proximal lateral restraint. (B) LPTL reconstruction illustrating a patella-to-tibia graft providing distal lateral stabilization. (C) Lateral retinacular reconstruction using a distally based ITB graft to augment the lateral retinacular layer. ITB, iliotibial band; LPFL, lateral patellofemoral ligament; LPTL, lateral patellotibial ligament.

Anatomic reconstructions represented the most frequently reported approach and were described in 14 studies^‖^ (Table 3), using heterogeneous graft sources (hamstring tendon autograft/allograft, quadriceps tendon, ITB) and patellar/femoral fixation strategies (transosseous tunnels, suture anchors, interference screws, cortical buttons), with both bone-tunnel and all-soft-tissue constructs described. Pedicled reconstructions preserving the native ITB attachment at Gerdy’s tubercle were reported in 5 studies,^2,8,9,21,31^ avoiding complete graft detachment and distal bone tunnels. Retinacular imbrication or sling-type repairs using fascia lata or native tissue were described in three studies^1,2,22^ and relied on soft-tissue or nonanatomic fixation; 2 studies^8,9^ described revision-specific hybrid constructions combining adjustable-loop cortical buttons with pedicled ITB augmentation (Figure 3).

**Table 3:** Surgical Technique Characteristics of LPFRC Reconstruction Including Graft Selection and Fixation Methods^*a*^

Study	Reconstruction Type	Graft Choice	Patellar Fixation	Femoral Fixation	Distal/Tibial Fixation	Technique Previously Described?
Abhaykumar et al^1^	Sling reconstruction	Fascia lata autograft	Transosseous lateral patellar drill hole	Looped under proximal fibular collateral ligament; graft sutured to itself	Native distal fascia lata attachment preserved at Gerdy’s tubercle	No
Aksahin et al^2^	Imbrication + augmentation	Pedicled IT band strip	Soft-tissue augmentation / no osseous patellar fixation	Not applicable (soft-tissue augmentation only)	Native ITB attachment preserved	Yes - Hughston et al. (1996)
Arora et al^4^	Anatomic reconstruction	Doubled peroneus longus autograft	Transosseous bone tunnel + Endobutton	Femoral tunnel with interference screw	—	No
Beckert et al^5^	Anatomic reconstruction	Semitendinosus allograft	Patellar soft-tissue suture fixation (FiberWire/Krackow)	Femoral tunnel with cortical Endobutton	—	No
Borbas et al^6^	Anatomic reconstruction	Free gracilis autograft	Transosseous patellar tunnels	Femoral tunnel with absorbable interference screw	—	No
Decilveo et al^9^ (2025a)	Anatomic reconstruction + augmentation	Double-limb semitendinosus allograft + ITB autograft	—	—	Pedicled ITB at Gerdy’s	Yes - Modified from Decilveo et al^8^ (2025b)
Decilveo et al^8^ (2025b)	Revision anatomic reconstruction + augmentation	Double-limb semitendinosus allograft + ITB autograft	Hybrid all-suture anchors	Adjustable-loop cortical button (TightRope II)	Pedicled ITB at Gerdy’s	No
Hughston et al^12^	Mixed repair/reconstruction techniques	ITB strip / patellar tendon strip / native tissue	Soft-tissue repair or patellar tunnel (subgroup dependent)	Soft-tissue or tibial fixation depending on subgroup	Tibial bone tunnel or soft-tissue fixation	No
Kerzner et al^13^	Anatomic soft-tissue reconstruction	Semitendinosus allograft	Soft-tissue onlay fixation	Femoral all-suture anchor	—	No
Moatshe et al^21^	Lateral patellotibial ligament reconstruction	ITB autograft + lateral patellar tendon strip	Patellar tendon strip harvested with patellar attachment preserved	None (soft-tissue–based reconstruction)	Native ITB attachment preserved at Gerdy’s tubercle	Yes, Hughston et al^12^; Sawyer et al^31^
Nonweiler et al^22^	Retinacular imbrication	Native lateral retinaculum	Soft-tissue imbrication	Soft-tissue repair	—	No
Parvaresh et al^23^	Combined ligament reconstruction	Hamstring allografts (2 grafts)	Soft-tissue fixation (no lateral bone tunnels)	Soft-tissue repair	—	No
Sanchis-Alfonso et al^26^ (2015a)	Anatomic reconstruction (deep transverse lateral retinaculum)	Anterior ITB strip autograft	Soft-tissue suture anchor	Soft-tissue fixation to ITB	—	No
Sanchis-Alfonso et al^27^ (2015b)	Anatomic reconstruction (deep transverse lateral retinaculum)	Anterior ITB strip autograft	Soft-tissue suture or anchor	Soft-tissue fixation to ITB	—	Yes, Sanchis-Alfonso et al^26^
Saper et al^28^	Anatomic reconstruction	Quadriceps tendon autograft (distally attached)	Native patellar attachment	Femoral suture anchor	—	No
Saper et al^30^ (2014a)	Anatomic reconstruction	Quadriceps tendon autograft (distally attached)	Native patellar attachment	Femoral suture anchor	—	Yes, Saper et al^30^
Saper et al^29^ (2014b)	Anatomic soft-tissue LPFL reconstruction	Double-bundled gracilis allograft	Subperiosteal/extensor retinacular tunnel over patella	Femoral tunnel with interference screw	—	No
Sawyer et al^31^	Anatomic LPTL reconstruction	Split patellar tendon+ IT band autograft	Soft-tissue fixation	None (soft-tissue–based construct)	IT band preserved at Gerdy’s tubercle	No
Teitge et al^35^	Anatomic reconstruction	Quadriceps tendon with bone block	Transverse patellar tunnel	Lag screw fixation at lateral epicondyle	—	No
Udagawa et al^36^	Combined revision reconstruction	Free gracilis autograft	Patellar bone tunnel with Endobutton fixation	Femoral tunnel with Endobutton CL fixation	—	Yes - Teitge et al^35^

a IT, iliotibial; ITB, iliotibial band; LPFL, lateral patellofemoral ligament; LPTL, lateral patellotibial ligament.

**Figure 3.**
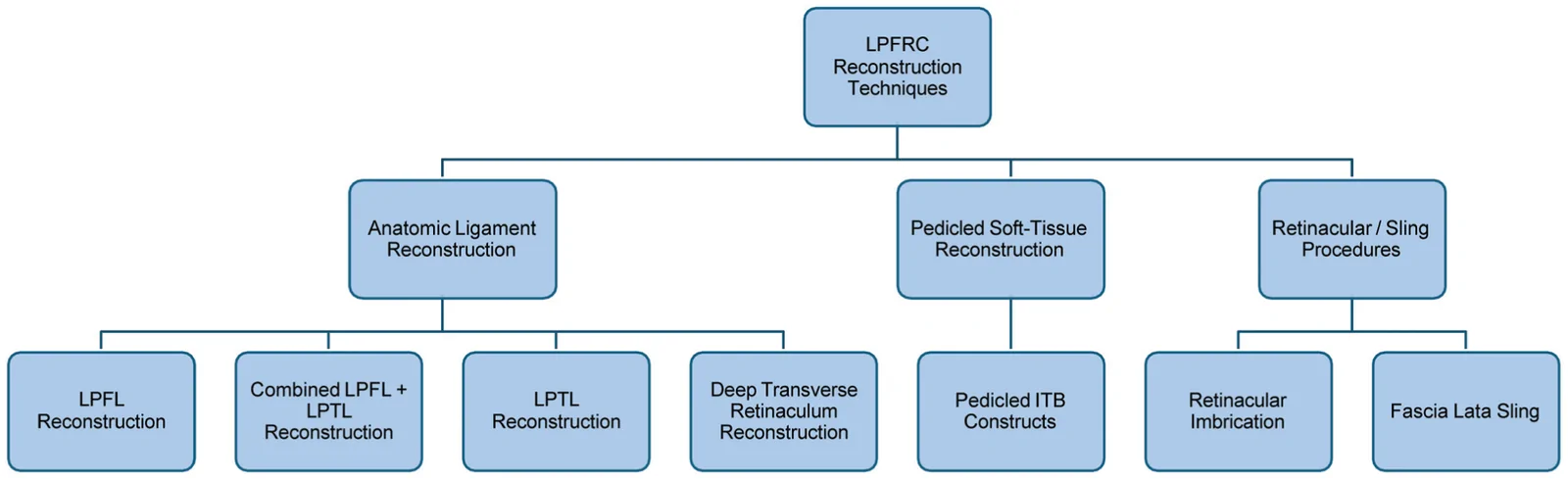
Classification of surgical techniques for LPFRC reconstruction. ITB, iliotibial band; LPFL, lateral patellofemoral ligament; LPFRC, lateral patellofemoral restraint complex; LPTL, lateral patellotibial ligament.

Across all techniques, no uniform graft type, fixation method, or configuration was adopted, and most constructs were presented as novel variations rather than reproductions of previously described techniques.

### Postoperative Rehabilitation Protocols

Rehabilitation protocols were reported with at least partial detail in 19 of 20 studies (Table 4) and varied considerably. Most studies (n = 16) used postoperative bracing, typically for 4 to 6 weeks with staged unlocking; 3 did not specify immobilization.^1,6,35^ Weightbearing ranged from immediate weightbearing as tolerated (10 studies)^¶^ to partial or restricted weightbearing for up to 6 weeks,^2,6,8,9,21,22,31^ generally progressing to full weightbearing by 4 to 6 weeks. Range-of-motion advancement similarly ranged from immediate mobilization to staged progression over 4 to 6 weeks when bracing was used. Strengthening and return-to-sport guidance, when reported, generally began at 6 to 12 weeks, with return to sport at approximately 3 to 6 months; criteria were largely surgeon-dependent, and no standardized rehabilitation protocol was consistently applied across studies.

**Table 4:** Postoperative Rehabilitation Protocols Following LPFRC Reconstruction^*a*^

Study	Immobilization	Weightbearing	ROM Progression	Strengthening/RTS
Abhaykumar et al^1^	None specified	Immediate WBAT	Immediate mobilization	Physical therapy initiated immediately
Aksahin et al^2^	Straight knee brace × 6 weeks	Partial WB × 4 weeks	Early ROM 0°-90°; full active by week 3	VL-focused strengthening × 3 months
Arora et al^4^	NR	NR	NR	NR
Beckert et al^5^	Hinged brace × 2 weeks	Immediate WBAT	Immediate ROM; goal 90° by 2 weeks	Early quadriceps activation
Borbas et al^6^	NS	Partial WB (15 kg) × 6 weeks	No ROM restriction reported	NR
Decilveo et al^9^ (2025a)	Hinged brace × 6 weeks	Restricted WB × 6 weeks	Early controlled ROM; advancing to full ROM as tolerated	Strengthening at 8-12 weeks
Decilveo et al^8^ (2025b)	Hinged brace × 6 weeks	Restricted WB × 6 weeks	Early controlled ROM; advancing to full ROM as tolerated	Strengthening at 8-12 weeks
Hughston et al^12^	Straight knee immobilizer × 3 days	Progressive WB	Early ROM within days	Gradual quadriceps strengthening
Kerzner et al^13^	Straight knee immobilizer× 2 weeks	WBAT	Staged progression (0°-30°, 0°-60°, 0°-90° over 6 weeks)	Plyometrics 14-22 weeks; RTS per surgeon
Moatshe et al^21^	Straight knee immobilizer × 6 weeks	Touch-down WB × 6 weeks	Passive ROM 0°-90° for first 2 weeks; full ROM as tolerated after week 2	Stationary bike + closed-chain strengthening after 6 weeks; return to full activity 4.5-6 months
Nonweiler et al^22^	Straight knee immobilizer× 6 weeks	Partial WB × 4 weeks; full at 4 weeks	0°-90° when out of brace	Quadriceps exercises at 4 weeks
Parvaresh et al^23^	Hinged brace × 6 weeks	Immediate WBAT	Immediate ROM	Closed-chain strengthening at 6weeks; RTS 3-4 months
Sanchis-Alfonso et al^26^ (2015a)	Straight knee immobilizer × 4-6 weeks	WBAT with crutches	Early flexion-extension from day 2	Brace off at 4 weeks
Sanchis-Alfonso et al^27^ (2015b)	Straight knee immobilizer × 4-6 weeks	WBAT with crutches	Early flexion-extension from day 2	Brace off at 4 weeks
Saper et al^28^	Straight knee immobilizer	WBAT × 2-4 weeks	Immediate ROM	Immediate quadriceps exercises
Saper et al^30^ (2014a)	Straight knee immobilizer	WBAT	ROM as tolerated	RTS at 4 months
Saper et al^29^ (2014b)	Straight knee immobilizer × 4-6 weeks	WBAT	0°-90° immediately	RTS at 4-6 months
Sawyer et al^31^	Straight knee immobilizer× 6 weeks	Touch-down WB × 6 weeks	0°-90° immediately (out of brace)	Gradual progression after 6 weeks
Teitge et al^35^	NS	Immediate full WB	Immediate full ROM	Immediate active exercises
Udagawa et al^36^	Straight knee immobilizer × 1 week	WB at 3 weeks	ROM initiated at 1 week	RTS at 6 months

a NR, not reported; NS, not specified; ROM, range of motion; RTS, return to sport; VL, vastus lateralis; WB, weightbearing; WBAT, weight bearing as tolerated.

### Clinical Outcomes

Clinical outcomes were reported in 10 studies (Tables 5A-5B). Outcome reporting formats varied: 6 studies reported numeric PROMs, 4 reported only categorical or narrative outcomes, and half of the outcome-bearing studies included cohorts of <5 patients.

**Table 5A:** Quantitative Clinical Outcomes^*a*^

Study	N, as Reported	Follow-up, Months	Recurrent Medial Instability	PROMs Reported	Preop Score	Postop Score	Mean Change
Beckert et al^5^	12 knees^*b*^	24.6	0/12	KOOS	34.39 (7.7-70.12)	69.54 (26.82-91.46); *P* < .0001	35.15
Decilveo et al^9^ (2025a)	1	24	0/1	Lysholm	22	81	59
Moatshe et al^21^	13	45.6 (24-60)	0/13	Lysholm; Tegner; WOMAC; IKDC	Lysholm: 45.6; Tegner: 2.9; WOMAC: 36.1; IKDC: 61	Lysholm: 71.9, *P* < .001; Tegner: 4.1, *P* < .17; WOMAC: 17.8, *P* < .02; IKDC: 66, *P* < .57	Lysholm: +26.30; Tegner: +1.20; WOMAC: –18.30; IKDC: +5
Nonweiler et al^22^	5	39.6 (12-84)	1/5^*c*^	Lysholm	48 (range, 33-65)	79 (45-90)	31
Sanchis-Alfonso et al^27^ (2015b)	17	56 ± 23.6 (24-96)	0/17	Lysholm; VAS	Lysholm: 36.4 ± 14.1; VAS: 7.6 ± 1.5	Lysholm: 86.1 ± 7.8; VAS: 1.9 ± 2.4; *P* < .001	Lysholm: +49.70; VAS: –5.70
Udagawa et al^36^	1	60	0/1	Kujala	69	93	24

a Data are presented as mean ± SD or mean (range). IKDC, International Knee Documentation Committee; KOOS, Knee injury and Osteoarthritis Outcome Score; Postop, postoperative; Preop, preoperative; PROMs, patient-reported outcome measures; VAS, visual analog scale; WOMAC, Western Ontario and McMaster Universities Osteoarthritis Index

b Beckert et al:^5^ KOOS values were reported for 12 knees with both pre- and postoperative scores available, rather than for the full patient cohort

c Nonweiler et al:^22^ One episode of “giving way” was reported; objective instability tests were negative.

**Table 5B:** Qualitative Clinical Outcomes^*a*^

Study	No. of Patients	Follow-up, Months	Recurrent Medial Instability	Outcome Reporting Type	Outcome Summary, As Reported
Abhaykumar et al^1^	3	6084	0/3	No PROM reported	Clinical improvement described; no numeric scores provided
Aksahin et al^2^	1	12	0/1	Categorical functional and pain scales	Hughston functional level and pain severity categories reported;^*b*^ no numeric scale values
Hughston et al^12^	63 (65 knees)	53.7 (24-99)	6/65 (~9%)	Categorical outcomes	Functional level categories; pain severity categories; subjective improvement rating
Saper et al^29^ (2014b)	3	12	0/3	No PROM reported	Objective stability tests negative; no numeric PROMs provided

a Data are presented as n, mean (range), or n/N (%). PROM, patient-reported outcome measure.

b Hughston pain scale: a categorical grading system assessing anterior knee pain severity based on activity level and functional limitation, ranging from no pain to pain with activities of daily living.

#### Quantitative Outcomes

Six studies reported numeric PROM data, with cohort sizes ranging from 1 to 17 patients (median, 8.5) and follow-up ranging from 24 to 60 months (median, 42.6 months). The Lysholm score was the most frequently used metric,^21,22,27^ with single studies each using the Kujala score,^36^ Knee injury and Osteoarthritis Outcome Score (KOOS),^5^ and additional measures (Tegner, Western Ontario and McMaster Universities Osteoarthritis Index, International Knee Documentation Committee, visual analog scale-pain). Postoperative scores improved relative to preoperative values across all studies reporting numeric comparisons (Table 5A).

#### Qualitative and Categorical Outcomes

Four studies^1,2,12,29^ reported outcomes using categorical functional or pain grading (Hughston criteria in 2 studies^2,12^), subjective improvement ratings, or stability testing rather than numeric scores (Table 5B). Across all outcome-bearing studies, 7 recurrent instability events were reported, concentrated in 2 studies; the remaining studies reported no recurrence at final follow-up, although inconsistent reporting formats and follow-up durations limit cross-study comparability.

### Complications

Complication reporting was inconsistent and generally sparse. Earlier series of retinacular repair or reconstruction reported stiffness, infection, synovitis, and reoperation,^1,12^ whereas several more recent case reports and small series reported no complications,^6,9,29,30,36^ a finding limited by small sample sizes and short follow-up. Technical reports typically described only theoretical risks (eg, graft failure, patellar fracture)^13,29,31,35^ rather than observed complications, precluding meaningful assessment of true complication rates. Accordingly, the reported postoperative improvements and infrequent recurrence should be interpreted as descriptive, hypothesis-generating findings from small, uncontrolled cohorts rather than evidence of treatment efficacy.

## Discussion

The primary findings of this systematic review are that LPFRC reconstruction for medial patellar instability has been described after prior lateral-sided surgical intervention, using a wide variety of grafts, fixation strategies, and construct designs. No single reconstructive approach was consistently adopted, and clinical outcome reporting was inconsistent.

The predominance of iatrogenic medial patellar instability likely reflects the historical use of aggressive lateral retinacular release for anterior knee pain and patellar maltracking; excessive release of the lateral retinaculum can disrupt the native lateral restraining structure of the patella, resulting in symptomatic medial subluxation or instability.^12,25^ Primary medial patellar instability without prior surgical intervention appears comparatively uncommon.^25^ This distribution underscores the importance of careful surgical decision-making when performing lateral-sided procedures and reinforces the role of the lateral restraints in maintaining patellofemoral stability.

A central observation of this review is the marked variation in surgical technique. Anatomic reconstructions were most commonly described, but graft selection, fixation methods, tensioning parameters, and construct design varied considerably, likely reflecting the rarity of medial patellar instability and the evolving understanding of the LPFRC. In the lateral patellar instability literature, MPFL repair has historically been associated with higher failure rates, contributing to a shift toward reconstruction.^3,7,17^ Over the past 2 decades, MPFL reconstruction has also converged toward consistent anatomic femoral fixation and standardized graft options.^19,32^ By contrast, LPFRC reconstruction remains less well defined and is often tailored to the suspected restraint deficiency or revision scenario.

Outcome reporting was limited and heterogeneous: fewer than half of the studies reported extractable numeric PROMs, and cohort sizes were generally small. Where reported, however, some improvements were substantial. Sanchis-Alfonso et al^27^ reported Lysholm scores improving from 36.4 ± 14.1 to 86.1 ± 7.8, a change exceeding commonly reported minimal clinically important difference (MCID) thresholds for the Lysholm score (approximately 8-10 points), and Beckert et al^5^ reported KOOS improvement from 34.39 to 69.54, likewise exceeding reported KOOS MCID thresholds (approximately 8-10 points),^24^ suggesting clinically meaningful improvements within these select cohorts despite the absence of comparative control groups. Recurrence of instability was reported infrequently, but given the small overall number of patients, the frequency of single-patient case reports, and variable follow-up durations, this low rate should be viewed as a preliminary observation rather than as evidence of long-term construct durability. More broadly, nearly half of the included studies were technical notes without associated patient cohorts, reflecting an imbalance between procedural innovation and outcome validation in this literature.

Previous reviews have primarily synthesized clinical outcomes and complications after surgical treatment.^20^ The present review instead examines the operative constructs themselves, including the targeted anatomic restraint, graft selection, fixation strategy, and reconstructive configuration, across both outcome-bearing and technical studies. This broader synthesis enabled organization of the literature into anatomic ligament reconstructions, pedicled soft-tissue reconstructions, and retinacular repair, imbrication, or sling-based procedures. This classification is not intended to serve as a validated treatment algorithm or to demonstrate the superiority of one strategy; rather, its value lies in distinguishing whether a procedure restores a defined ligament, preserves a pedicled native attachment, or relies on soft-tissue repair or nonanatomic restraint, providing standardized terminology for comparing procedures and relating construct selection to the suspected pattern of lateral restraint deficiency.

Although the available evidence does not permit definitive indications, isolated LPFL reconstruction may be considered when symptomatic medial instability is believed to arise predominantly from insufficiency of the femoropatellar component and the distal lateral restraints remain functionally intact. Conversely, combined LPFL/LPTL reconstruction may be considered when there is more extensive LPFRC deficiency, instability across a broader arc of knee flexion, or persistent or recurrent instability after prior lateral-sided reconstruction or extensive lateral release. This rationale is anatomically plausible because the LPFL and LPTL exhibit distinct flexion-dependent length-change patterns, suggesting complementary restraint throughout knee flexion.^10,11^ However, combined reconstruction was reported in only 2 revision-focused publications in this review,^8,9^ and no comparative study has established its superiority over isolated reconstruction. Accordingly, the proposed classification should guide procedural description and hypothesis generation rather than dictate surgical selection.

These findings should be interpreted in light of several limitations. The available literature consisted predominantly of Level 4 and 5 evidence, with no comparative or randomized studies identified, and outcome-bearing cohorts were small and potentially overlapping. PROMs, follow-up durations, and rehabilitation protocols were inconsistently reported, limiting cross-study comparability and precluding pooled quantitative analysis. Publication bias may be present, as technically successful cases may be more likely to be reported than complications or failures. Future investigations should prioritize prospective, multicenter or registry-based cohort development with standardized reporting of surgical technique, fixation parameters, and validated outcome measures to enable meaningful comparative evaluation as the evidence base matures.

## Conclusion

Reconstruction of the LPFRC for medial patellar instability is supported by a technically diverse but methodologically limited body of literature, most commonly arising in the setting of prior lateral-sided intervention. Although recurrence events were infrequently reported, the predominance of small observational cohorts and technical descriptions limits confident assessment of construct-specific effectiveness and durability. The present review highlights a clear imbalance between procedural innovation and systematic outcome validation. Future investigation should prioritize prospective cohort development, standardized reporting of surgical constructs and tensioning parameters, and consistent use of validated outcome measures to enable meaningful comparative evaluation as the evidence base evolves.

## Footnotes

§ References 4, 5, 6, 8, 9, 13, 23, 28, 29, 30, 35.

‖ References 4, 5, 6, 8, 9, 13, 26, 27, 28, 29, 30, 31, 35, 36.

¶ References 1, 5, 13, 23, 26, 27, 28, 29, 30, 35.

## Appendix

**Supplementary Material 1:** Line-by-line Search Strategies for Each Database^*a*^ *Ovid MEDLINE(R) ALL <1946 to Jan 09, 2026>

No.	Query	Results From 11 January 2026
1	exp Patellofemoral Joint/	1858
2	(patella* adj3 (instabilit* or dislocat* or sublux* or maltrack* or malalign* or shift*)).ti,ab,kw.	2501
3	(patellofemoral adj3 (instabilit* or dislocat* or sublux* or maltrack* or malalign* or pain)).ti,ab,kw.	4909
4	(patellofemoral adj3 (instabilit* or dislocat* or sublux* or maltrack* or malalign* or pain)).ti,ab,kw.	3684
5	1 or 2 or 3 or 4	8932
6	(lateral patellofemoral* or LPFL or "lateral patellofemoral ligament*" or "lateral patellofemoral restraint*" or "lateral patellofemoral complex").ti,ab,kw.	316
7	(lateral retinacul* or lateral retinacular or "lateral retinaculum").ti,ab,kw.	627
8	(lateral capsul* adj3 (knee or patell* or femor*)).ti,ab,kw.	14
9	(patell* adj3 (lateral ligament* or lateral restraint* or lateral tether* or lateral stabiliz*)).ti,ab,kw.	9
10	6 or 7 or 8 or 9	921
11	exp Reconstructive Surgical Procedures/	279908
12	exp Orthopedic Procedures/	396317
13	(reconstruct* or repair* or surg* or operat* or augment* or graft* or tenodesis or fixation).ti,ab,kw.	4794883
14	exp Surgical Procedures, Operative/	3789748
15	11 or 12 or 13 or 14	6852903
16	5 and 10 and 15	457
*Ovid EMBASE <1974 to Jan 8, 2026>		
No.	Query	Results from 11 Jan 2026
1	exp patella dislocation/	4271
2	exp patellofemoral joint/	6537
3	(patella* adj3 (instabilit* or dislocat* or sublux* or maltrack* or malalign* or shift*)).ti,ab.	5634
4	(patellofemoral adj3 (instabilit* or dislocat* or sublux* or maltrack* or malalign* or pain)).ti,ab.	4600
5	1 or 2 or 3 or 4	14275
6	(lateral patellofemoral* or LPFL or "lateral patellofemoral ligament*" or "lateral patellofemoral restraint*" or "lateral patellofemoral complex").ti,ab.	398
7	(lateral retinacul* or lateral retinacular or "lateral retinaculum").ti,ab.	702
8	(lateral capsul* adj3 (knee or patell* or femor*)).ti,ab.	16
9	(patell* adj3 (lateral ligament* or lateral restraint* or lateral tether* or lateral stabiliz*)).ti,ab.	6
10	6 or 7 or 8 or 9	1073
11	exp surgery/	6888074
12	exp reconstructive surgery/	251156
13	exp orthopedic surgery/	697869
14	(reconstruct* or repair* or surg* or operat* or augment* or graft* or tenodesis or fixation).ti,ab.	6323493
15	11 or 12 or 13 or 14	9571198
16	5 and 10 and 15	574
*SCOPUS <1778 to Jan 11, 2026>		
#	Query	Results from 11 January 2025
1	TITLE-ABS-KEY( patella* W/3 (instabilit* OR dislocat* OR sublux* OR maltrack* OR malalign* OR shift*) OR patellofemoral W/3 (instabilit* OR dislocat* OR sublux* OR maltrack* OR malalign* OR pain) )	7627
2	TITLE-ABS-KEY( "lateral patellofemoral*" OR LPFL OR "lateral patellofemoral ligament*" OR "lateral patellofemoral restraint*" OR "lateral patellofemoral complex" OR "lateral retinacul*" OR "lateral retinaculum" OR "lateral retinacular" OR (lateral W/3 (capsul* OR ligament* OR restraint* OR tether* OR stabiliz*) W/3 patell*) )	1529
3	TITLE-ABS-KEY( reconstruct* OR repair* OR surg* OR operat* OR augment* OR graft* OR tenodesis OR fixation )	13489512
4	#1 and #2 and #3	613
*Web of Science <1637to Jan 11, 2026>		
No.	Query	Results From 11 January 2026
1	TS=( patella* NEAR/3 (instabilit* OR dislocat* OR sublux* OR maltrack* OR malalign* OR shift*) OR patellofemoral NEAR/3 (instabilit* OR dislocat* OR sublux* OR maltrack* OR malalign* OR pain) )	12650
2	TS=( "lateral patellofemoral*" OR LPFL OR "lateral patellofemoral ligament*" OR "lateral patellofemoral restraint*" OR "lateral patellofemoral complex" OR "lateral retinacul*" OR "lateral retinaculum" OR "lateral retinacular" OR (lateral NEAR/3 (capsul* OR ligament* OR restraint* OR tether* OR stabiliz*) NEAR/3 patell*) )	2821
3	TS=( reconstruct* OR repair* OR surg* OR operat* OR augment* OR graft* OR tenodesis OR fixation )	29494597
4	1 AND 2 AND 3	720

a LPFL, lateral patellofemoral ligament.

**Supplementary Material 2:** MINORS Score^*a*^

Study	Design (Comparative/ NonComparative)	1. Aim	2. Consecutive Patients	3. Prospective Data	4. Appropriate Endpoints	5. Unbiased Endpoint assessment	6. Adequate Follow-up	7. < 5% Loss to Follow-up	8. Prospective Sample Size Calculation	9. Adequate Control Group	10. Contemporary Groups	11. Baseline Equivalence of Groups	12. Adequate Statistical Analyses	Total Score
Abhay kumar et al^1^	Noncomparative	2	0	0	1	0	2	1	0	N/A	N/A	N/A	N/A	6
Beckert et al^5^	Noncomparative	2	2	0	2	0	1	1	0	N/A	N/A	N/A	N/A	8
Hughston et al^12^	Noncomparative	2	2	0	2	0	2	1	0	N/A	N/A	N/A	N/A	9
Nonweiler et al.	Noncomparative	2	1	0	2	0	2	2	0	N/A	N/A	N/A	N/A	9
Sanchis- Alfonsoetal.	Noncomparative	2	2	0	2	0	2	2	0	N/A	N/A	N/A	N/A	10
Moatshe et al^21^	Noncomparative	2	2	2	2	0	2	1	0	N/A	N/A	N/A	N/A	11

a MINORS, Methodological Index for Non-Randomized Studies; N/A, not applicable.

**Supplementary Material 3:** Qualitative Breakdown of Papers^*a*^

Study	Study Type	Patient Selection Clearly Described	Indication for Surgery Clearly Reported	Operative Technique Detail (Graft, Fixation, Tensioning)	Outcome Reporting Quality	Follow-up Duration Reported	Complication Reporting	Rehabilitation Protocol Reported	Overall Methodological Comment
		Yes - single 21-year-old patient with spontaneous medial patellar instability	Yes - persistent symptoms despite	Detailed–IT band flap used for patellotibial ligament				Yes - brace + progressive ROM	Well-described case with detailed surgical technique but
Aksahin et al^2^	Case report	described	physical therapy	Augmentation	Narrative clinical outcomes only	Yes - postoperative follow-up described	No complications reported	program described	Limited generalizability due to single patient
		Yes - 62-year-old patient with medial patellar	Yes - symptomatic instability after prior					Brief postoperative protocol	Clear surgical technique description with limited outcome
Borbas et al^6^	Case report	Instability after TKA	procedures	Detailed–LPFL reconstruction with gracilis autograft	Clinical and radiologic outcomes at 1 year	Yes - 1-year follow-up	No complications reported	described	Reporting typical of case reports
		Yes - 35-year-old patient with recurrent		Detailed–revision LPFL reconstruction with semitendinosus			No postoperative complications		High technical detail and longer follow-up, but still limited
DeCilveo et al^9^	Case report	Iatrogenic medial instability	Yes - failure of prior reconstruction	Allograft + ITB augmentation	PROM reported (Lysholm improvement)	Yes - 2-year follow-up	reported	Rehabilitation described	By single-patient design
		Yes - young female with bilateral instability	Yes - recurrent instability after MPFL					Postoperative protocol briefly	Good long-term follow-up but evidence level limited by
Udagawaetal^36^	Case report	After prior surgery	Reconstruction + lateral release	Detailed–MPFL revision + lateral retinacular reconstruction	Functional score improvement (Kujala) reported	Yes - 5-year follow-up	No major complications reported	described	Case report design
		Yes - 14-year-old female with medial patellar	Yes - symptomatic medial patellar instability	Detailed–LPFL reconstruction using quadriceps tendon graft with ITB flap augmentation and tibial tubercle			No postoperative complications	Yes - partial weightbearing with	Detailed operative description and postoperative course; however, limited external validity due to single-patient
Saper& Shn^30^ eider	Case report	Subluxation without prior lateral release	With pain and functional limitation	transfer	Narrative clinical outcome with complete symptom resolution	Follow-up reported but exact duration not specified	Ied reported	Progressive ROM and strengthening	design

a ITB, iliotibial band; LPFL, lateral patellofemoral ligament; MPFL, medial patellofemoral ligament; PROM, patient-reported outcome measure; ROM, range of motion; TKA, total knee arthroplasty.
